# Hyperferritinemia in children hospitalized with scrub typhus

**DOI:** 10.1186/s41182-021-00304-4

**Published:** 2021-02-17

**Authors:** Vijai Williams, Nisha Menon, Prateek Bhatia, Manisha Biswal, Sreejesh Sreedharanunni, Muralidharan Jayashree, Karthi Nallasamy

**Affiliations:** 1 Division of Pediatric Emergency and Intensive care, Department of Pediatrics, Advanced Pediatrics Centre, Postgraduate Institute of Medical Education & Research, Sector-12, Chandigarh, 160012 India; 2 Division of Pediatric Hematology, Department of Pediatrics, Advanced Pediatrics Centre, Postgraduate Institute of Medical Education & Research, Chandigarh, India; 3 Department of Medical Microbiology, Postgraduate Institute of Medical Education & Research, Chandigarh, India; 4 Department of Hematology, Postgraduate Institute of Medical Education & Research, Chandigarh, India

**Keywords:** Pediatric, Ferritin, Scrub typhus, Mortality, Sepsis

## Abstract

**Background:**

Hyperferritinemia is increasingly associated with mortality in sepsis. Studies estimating the prevalence of hyperferritinemia in pediatric scrub typhus are limited.

**Methods:**

This was a secondary analysis of a prospective observational study (FERRIS) from a tertiary care teaching hospital in North India where 72 children with confirmed scrub typhus, 4 (5.5%) PCR positive, 55 (76.4%)-IgM ELISA positive, and 13 (18.1%)-both PCR and ELISA positive, were analyzed. Serum ferritin was measured in 62 children to identify the prevalence of hyperferritinemia and determine its association with mortality.

**Results:**

Hyperferritinemia (> 500 μg/L) was seen in 72.6% [*n* = 45] children; 26 (41.9%) were mild (500–2000 μg/L), 13 (21%) were moderate (2000–10,000 μg/L), and 6 (9.7%) were severe (> 10,000 μg/L). Early presentation to hospital (≤ 7 days of febrile illness) had more survivors than late presentation (> 7 days). Non-survivors had significantly higher PRISM III, PELOD-2, hyperlactatemia, hypoalbuminemia, organ dysfunction, need for mechanical ventilation, and need of RRT. Ferritin had poor sensitivity and specificity in predicting survival with AUC of 0.56. Organ dysfunction and risk scores as PRISM III, PELOD 2, and VIS at admission were better predictors with AUC (95% CI) of 0.72 (0.56, 0.89), 0.77 (0.63, 0.92), and 0.90 (0.78, 1.0) respectively.

**Conclusions:**

Hyperferritinemia is common in scrub typhus but it did not predict survival. Organ dysfunction and risk scores were better predictors of mortality than ferritin.

## Background

Scrub typhus is a common cause of acute undifferentiated fever in Asian countries [1]. The disease manifests with protean clinical features varying from undifferentiated fever to several organ dysfunctions that include acute respiratory distress syndrome (ARDS), myocarditis, acute kidney injury (AKI), encephalitis, acute liver failure, and culminating in multiorgan dysfunction syndrome (MODS) [2, 3]. Early suspicion and treatment are essential as delay increases morbidity and mortality multifold. A significant overlap of symptoms with other viral and certain bacterial sepsis makes the diagnosis difficult, and hence, a syndromic approach is recommended [4]. The pathogenesis differs from other bacterial causes of sepsis in early and severe involvement of vascular endothelium [5]. Hematological dysfunction is prominent; most present with thrombocytopenia as a prominent feature with varying degrees of anemia [6]. Other organ dysfunctions are not uncommon, and they have an additive association with mortality [7–9].

Several clinical and laboratory parameters showed inconsistent results in predicting organ dysfunction and survival in pediatric scrub typhus [9–11]. Ferritin can be an attractive marker as hematological dysfunction is prominent in these children especially in those with MODS [12]. From a mere marker of iron stores, the role of ferritin in disease pathogenesis and immunomodulation is increasingly being explored in sepsis [12–14]. Hyperferritinemic syndrome seen in critically ill children is attributed secondary to hypercytokinemia and macrophage activation, both well-known in scrub typhus [15]. However, robust studies on ferritin measurements in this cohort are lacking.

We hypothesized that early elevated ferritin may help identify the severe cases of scrub typhus that progress to MODS and eventual death. Hence, we planned this observational study to measure serum ferritin in children with confirmed scrub typhus and determine its association with disease severity and outcome.

## Methods

This was a secondary analysis of the ferritin in sepsis (FERRIS) study [16] which was a single center, prospective study at a tertiary care teaching hospital in North India. Children aged between 1 month and 12 years admitted to Pediatric Emergency Department and Pediatric Intensive Care Unit (PICU) of the Advanced Pediatrics Center, Postgraduate Institute of Medical Education and Research, were consecutively enrolled from July 2019 to December 2019 (6 months), the season with peak incidence of tropical infections. We chose this age cutoff as the upper age limit for pediatric admission is 12 years as per hospital policy.

Children with acute febrile illness (fever > 38.3 °C for more than 48 h duration and onset < 14 days) with either (a) cytopenia (platelet count < 100,000/mm^3^, and/or hemoglobin < 10 g/dl), (b) organomegaly (hepatomegaly/splenomegaly), (c) lymphadenopathy, or (d) systemic signs (rash, edema) were screened for eligibility and included if they were confirmed scrub typhus either by PCR or IgM ELISA. Children who received a transfusion were excluded. Less severe illness with presentation as undifferentiated fever without organ dysfunction and those who did not require hospitalization were not included in this study.

Children who met the eligibility criteria were enrolled consecutively after obtaining a written informed consent. Demographic, clinical, and laboratory data were collected. Complete blood counts and renal and liver function tests were performed at presentation for all cases and were repeated if necessary. Acute kidney injury was staged as per KDIGO guidelines [17]. Treatment-related variables, severity score (PRISM III, PELOD2), details of organ supportive therapies, length of PICU stay, and hospital outcome were recorded.

Diagnosis of scrub typhus was confirmed by PCR and/or IgM ELISA. Nested PCR was performed using the DNA extracted from the whole blood and was amplified to detect *Orientia tsutsugamushi* DNA. We used the oligonucleotide primers that were based on the nucleotide sequences of a gene encoding for the 56-kDa antigen of a Gilliam strain of *Orientia tsutsugamushi*. ELISA for scrub typhus IgM antibody was performed using Scrub Typhus Detect IgM ELISA (InBios International Inc., Seattle, WA). An optical density of ≥ 0.5 was considered as diagnostic for scrub typhus. All children underwent blood culture, Dengue IgM ELISA, NS1 antigen ELISA, IgM for leptospirosis, blood smears for malaria, Widal test, and serology for hepatitis A and E to rule out other seasonal infections.

Serum ferritin was estimated from the 2–3 ml EDTA blood sample collected at admission. Plasma was separated after centrifugation at 3000 rpm for 15 min and stored at – 80 °C. Samples were processed in batches of 10–15 samples every week using the chemiluminescence principle on the ADVIA Centaur Ferritin System (Siemens Healthcare Diagnostics, Los Angeles, CA) as per manufacturer’s instructions. If clinical suspicion of hemophagocytic lymphohistiocytosis (HLH) (persistence of fever, worsening cytopenia, and organ dysfunction) and/or elevated serum ferritin ≥ 2000 μg/L, then other relevant tests such as triglycerides, fibrinogen, and/or bone marrow examination were performed as per clinician’s decision. Decision to treat HLH with immunomodulatory therapy (steroids, IvIg, or both) was at the treating clinician’s discretion. The study protocol was approved by the Institute Ethics Committee.

### Definitions

Hyperferritinemia was defined as ferritin levels more than 500 μg/L [12, 15, 18]. We stratified hyperferritinemia into mild (500–2000), moderate (> 2000–10,000), and severe (> 10,000) categories. We classified the subjects into early presenters (≤ 7 days of febrile illness) and late presenters (> 7 days of febrile illness) to see if day of presentation since fever onset affected degree of hyperferritinemia. Organ dysfunction was defined as per Pediatric Logistic Organ Dysfunction score (PELOD-2) [19]. PELOD-2 was done daily till 5 days or till discharge which ever was earlier. The vasoactive drugs used during therapy were monitored and daily vasoactive inotrope score (VIS) was calculated [20].

### Outcome

The primary outcome was to identify the prevalence of hyperferritinemia in hospitalized children with scrub typhus. The secondary outcomes were to study the predictors of mortality in scrub typhus and determine the association of serum ferritin levels with duration of illness and mortality.

### Statistical analysis

Descriptive statistics including frequency, mean, median, interquartile range (IQR), and standard deviation (SD) were calculated for the demographic data and laboratory parameters. The prevalence of hyperferritinemia (> 500 ng/ml) reported from previous studies was 25–55% [13, 15]. Hence, we estimated sample size as 70 based on the assumed prevalence estimate of 25%, with a precision of 10% and 95% confidence interval (CI). Comparison of characteristics between survivors and nonsurvivors was made with Chi-square test for categorical data and the Mann-Whitney test for continuous nonparametric data. Predictors of mortality were identified using binary logistic regression. The sensitivity and specificity for different cutoff values of ferritin were analyzed using a receiver operating characteristic (ROC) curve and were compared with organ dysfunction and risk scores. For all tests, a two-sided *P* value of < 0.05 was considered statistically significant. All statistical analyses were performed using the SPSS software version 23.0 (SPSS Inc., Chicago, IL).

## Results

### Baseline characteristics

**Fig. 1:**
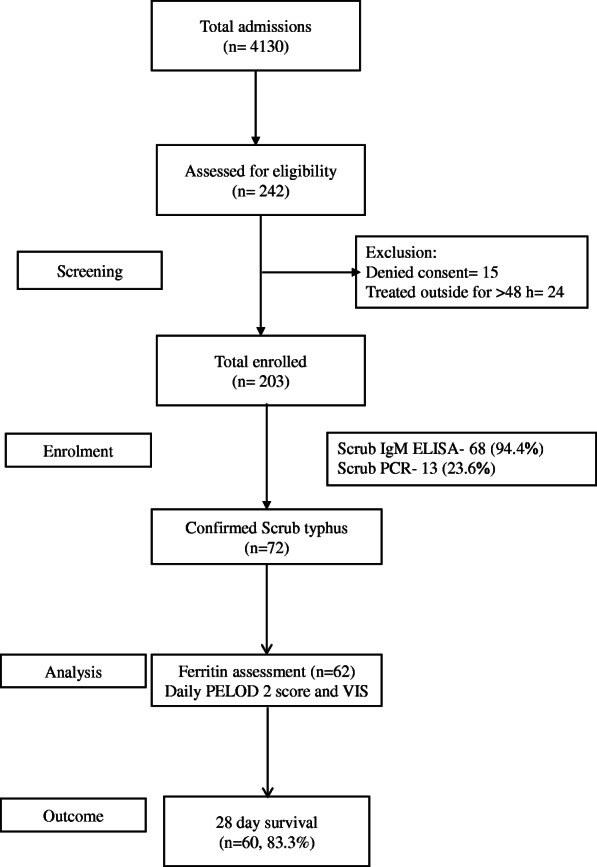
Study flow diagram

During the 6 months of study period, 10,158 children visited Pediatric Emergency Department and 4130 of them required admission. A total of 242 children were eligible, and only 72 children met the inclusion criteria (Fig. 1). Of them, 4 were (5.5%) PCR positive, 55 (76.4%)-IgM ELISA positive, and 13 (18.1%)-both PCR and ELISA positive. The median (IQR) age of presentation was 60 (36, 96) months with a male preponderance (*n* = 45, 62.5%) (Table 1). The median (IQR) PRISM III and PELOD 2 score at admission were 8 (5, 12) and 3 (2, 7) respectively. Fever was present in all; generalized edema, fast breathing, and abdominal pain were other common symptoms. Hepatomegaly was seen in 66 (91.7%) and splenomegaly in 43 (59.7%) children. Eschar, a pathognomonic clue to trombiculid bite, was seen only in 14 (19.4%) children. A rash was noted in 11% patients which varied from macular, blanching erythematous rash to petechial purpuric lesions.

**Table 1:** Clinical profile, investigations, and outcomes

Parameter	***N*** = 72
Age in months	60 (36, 96)
Boys, *n* (%)	45 (62.5)
Weight in kg	16.0 (11.4, 20.75)
Weight in *Z* score	− 1.37 (− 2.18, − 0.24)
PRISM III	8 (5, 12)
Duration of illness in days	7 (5, 10)
**Clinical features**	
Fever, *n* (%)	72 (100)
Edema, *n* (%)	35 (48.6)
Fast breathing, *n* (%)	27 (37.5)
Abdominal pain, *n* (%)	25 (34.7)
Bleeding manifestations, *n* (%)	17 (23.6)
Altered sensorium, *n* (%)	16 (22.2)
Seizures, *n* (%)	16 (22.2)
Headache, *n* (%)	14 (19.4)
Purpuric rash, *n* (%)	8 (11.1)
Oliguria, *n* (%)	6 (8.3)
Hepatomegaly, *n* (%)	66 (91.7)
Splenomegaly, *n* (%)	43 (59.7)
Eschar, *n* (%)	14 (19.4)
**Investigations**	
Hemoglobin, g/dl	8.7 (7.4, 10.2)
Platelet count, cells/mm^3^	44,000 (18,000, 80,000)
Total bilirubin, mg/dl	0.8 (0.6, 1.9)
Aspartate transaminase, U/L	185 (94, 285)
Alanine transaminase, U/L	79 (49, 125)
International normalized ratio	1.2 (1.0, 1.5)
**Treatment**	
Doxycycline, *n* (%)	65 (90.3)
Azithromycin, *n* (%)	7 (9.7)
IvIg only, *n* (%)	2 (2.8)
IvIg with steroid, *n* (%)	2 (2.8)
**Outcome**	
Survivors, *n* (%)	60 (83.3)
Length of PICU stay, days	5 (4, 9)
Length of hospital stay, days	5 (3, 8)

All values expressed as median (IQR)

*PRISM* Pediatric Risk of Mortality

The median (IQR) hemoglobin was 8.7 (7.4, 10.2) g/dL (Table 2). The median (IQR) platelet count was 44,000 (18,000, 80,000) cells/mm^3^. Severe hypoalbuminemia was common; more than half had serum albumin < 2.5 g/dL at admission. Elevated liver enzymes (> 5 times elevation of AST/ ALT) were seen in 27 of 65 children (41.5%).

**Table 2:** Comparison of baseline characteristics among survivors and nonsurvivors

	***N***	Total (***n*** = 72)	Survivors (***n*** = 60)	Nonsurvivors (***n*** = 12)	***P*** value
**Parameter**					
Age, months	72	60 (36, 96)	60 (30, 98)	36 (31, 105)	0.60
Boys, *n* (%)	72	45 (62.5)	41 (68.3)	4 (33.3)	**0.04**
PRISM III	72	8 (5, 12)	8 (5, 11)	12 (8.7, 17.5)	**0.005**
Duration of illness, days	72	7 (5, 10)	7 (5, 9.5)	9 (5, 12)	0.38
**Investigations**					
Ferritin, μg/L	62	668 (420, 2714)	666 (382, 2653)	1806 (443, 4727)	0.53
Normal (< 500), *n* (%)		17 (27.4)	15 (28.8)	2 (20)	0.42
Mild (> 500–2000), *n* (%)		26 (41.9)	23 (44.2)	3 (30)	
Moderate (> 2000–10,000), *n* (%)		13 (21)	10 (19.2)	3 (30)	
Severe (> 10,000), *n* (%)		6 (9.7)	4 (7.8)	2 (20)	
Hemoglobin, g/dl	72	8.7 (7.4, 10.2)	8.6 (7.5, 10.5)	8.1 (6.8, 10.1)	0.48
Total leukocyte count cells/mm^3^	72	14,000 (9820, 18,000)	14,090 (10,200, 17,500)	12465 (7020, 21,080)	0.39
Platelet count cells/mm^3^	72	18,000 (44,000, 80,000)	19,000 (48,000, 80,000)	31000 (11,500, 100,000)	0.39
Albumin, g/dl	58	2.2 (2.1, 2.6)	2.3 (2.1, 2.6)	2.1 (1.9, 2.2)	**0.05**
Severe hypoalbuminemia (< 2.5 g/dl), *n* (%)	58	39 (54.2)	30 (50)	9 (75)	0.14
Lactate, mmol/L	72	1.6 (1.1, 2.6)	1.6 (1, 1.8)	3.0 (1.85, 9.8)	**0.04**
**Organ dysfunction**					
Hematological dysfunction, *n* (%)	72	68 (94.4)	56 (93.3)	12 (100)	0.60
Respiratory failure,* n* (%)	72	40 (55.5)	28 (46.6)	12 (100)	**0.001**
ARDS, *n* (%)	72	23 (31.9)	13 (21.7)	10 (83.3)	**< 0.0001**
Mechanical ventilation, *n* (%)	72	34 (47.2)	22 (36.7)	12 (100)	**0.0001**
Shock, *n* (%)	72	20 (28.2)	12 (20.3)	8 (66.7)	**0.003**
VIS on day 1	72	0 (0, 10)	0 (0,0)	10 (0,30)	**0.0001**
AKI, *n* (%)	72	15 (20.8)	10 (16.7)	5 (41.7)	**0.02**
RRT, *n* (%)	72	5 (6.9)	2 (3.3)	3 (25)	**0.03**
CNS dysfunction, *n* (%)	72	10 (13.9)	6 (10)	4 (33.3)	**0.05**
Coagulopathy (INR > 1.5), *n* (%)	56	12 (21.4)	10 (21.3)	2 (22.2)	1.0
PELOD-2 score					
Day 1	72	3 (2, 7)	2 (2, 5)	7 (7, 9)	**< 0.0001**
Day 2	67	2 (1, 5)	2 (1, 5)	6 (4, 7)	**0.001**
Day 3	64	2 (1, 5)	2 (1, 4)	4 (1, 6)	0.32

All values expressed as median (IQR)

*PRISM* Pediatric Risk of Mortality, *PELOD* Pediatric Logistic Organ Dysfunction, *ARDS* Acute Respiratory distress syndrome, *VIS* vasoactive inotrope score, *AKI* acute kidney injury, *RRT* renal replacement therapy

### Clinical course

A total of 36 (50%) children required PICU admission. Hematological dysfunction was the commonest organ dysfunction [*n* = 68, 94.4%]. Respiratory failure [*n* = 40, 56%] was the next common organ dysfunction; acute respiratory distress syndrome (ARDS) was seen in 31.9% children. Nearly half (*n* = 34, 47%) received mechanical ventilation. Twenty children (28.2%) had shock requiring vasoactive drug support. AKI was seen in 15 (20.8%) children, and 5 (7%) required RRT. Encephalopathy with GCS < 10 was seen in 10 (13.9%) children. Sixty-five children were treated with doxycycline, and 7 received azithromycin. The median (IQR) length of hospital stay was 5 (3, 8) days. Sixty children survived to hospital discharge, and all [*n* = 60, 83.3%] were alive at 28-day follow-up. Four children (5.5%) had clinical features and investigations that fulfilled HLH diagnostic criteria. All 4 received intravenous immunoglobulin (IvIg), and 2 of them were also treated with methylprednisolone. Of them, 2 survived and 2 died.

### Hyperferritinemia

Ferritin estimation was possible in 62 children. Ferritin measurement of 10 samples was disregarded owing to inadequate or improper sample and hemolysis during storage. The median (IQR) day of ferritin measurement since symptom onset was 7 (5, 10) days. The median (IQR) ferritin level was 668 (42, 2714) μg/L. Seventeen children (27.4%) had ferritin in normal range. Hyperferritinemia (> 500 μg/L) was seen in 45 (72.6%) children; 26 (41.9%) were mild, 13 (21%) were moderate, and 6 (9.7%) were severe. Ferritin levels were not significantly different between survivors and nonsurvivors (*p* = 0.53).

**Fig. 2:**
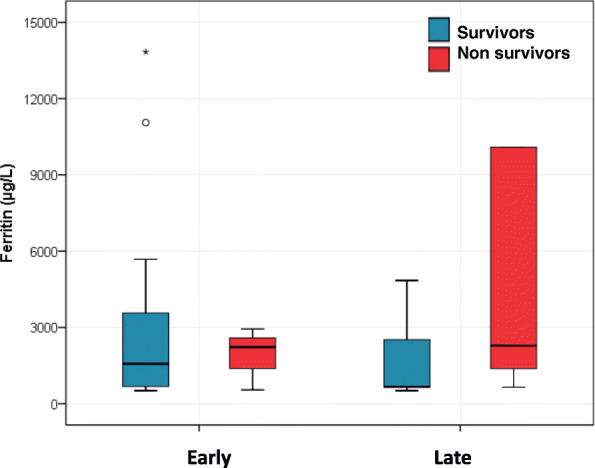
Boxplot showing differences in median ferritin value between early and late presentation

Among early presenters, 25 children had hyperferritinemia of whom 22 (88%) survived. Of late presenters, 20 children had hyperferritinemia and 15 (75%) survived. This difference did not reach statistical significance [*p* = 0.2, OR (95%) 2.4 (0.5, 11.4)]. The difference in ferritin levels between survivors and nonsurvivors at these two timelines was also not significant (Fig. 2).

### Predictors of outcome

**Fig. 3:**
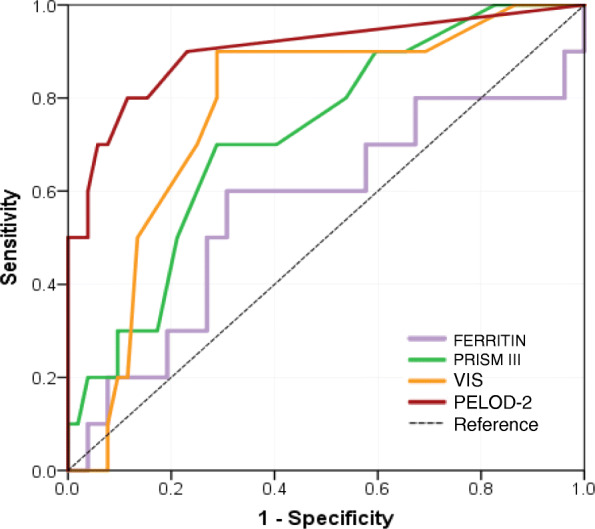
Receiver operating characteristic curve comparing ferritin, PELOD 2 score, and VIS for predicting mortality

Although nonsurvivors had significantly higher PRISM III, PELOD-2, hyperlactatemia, hypoalbuminemia, organ dysfunction, need for mechanical ventilation, and need of RRT, on binary logistic regression analysis, none of them was shown to predict mortality. Ferritin was higher in nonsurvivors [1806 (443, 4727) μg/L] than survivors [666 (382, 2653) μg/L], but the difference was statistically not significant. We found that ferritin has poor sensitivity and specificity in predicting mortality with AUC (95% CI) of 0.56 (0.35, 0.78) (Fig. 3). It was observed that higher ferritin values increased specificity to predict mortality. Ferritin value above 496 μg/L had 80% sensitivity and 29% specificity, and value above 2000 μg/L had 50% sensitivity and 73% specificity while value above 7880 μg/L had 20% sensitivity and 93% specificity. We found that organ dysfunction and risk score as PRISM III, PELOD 2, and VIS at admission were better predictors with AUC (95%CI) of 0.72 (0.56, 0.89), 0.77 (0.63, 0.92), and 0.90 (0.78, 1.0) respectively. VIS score > 16.5 was 80% sensitive and 88% specific to predict mortality. A PELOD-2 score > 7 had 80% sensitivity and 91% specificity while a PRISM III score > 10 had 70% sensitivity and 71% specificity in predicting mortality.

## Discussion

In this prospective study, we found that hyperferritinemia is common in scrub typhus and about a third (30.7%) had ferritin values more than 2000 μg/L. Severe hyperferritinemia (> 10,000 μg/L) was seen in 10%. Ferritin did not have good predictive ability for outcome though the nonsurvivors had higher proportion of moderate and severe hyperferritinemia than survivors. PELOD-2 and VIS score fared better than ferritin in predicting mortality. The reported case fatality rate in scrub typhus varies between 1.5 and 30% with a median of about 8% [2, 21]. The case fatality rate in this study is higher (16.7%). This may be due to the referral bias and the inclusion criteria selecting cases with high severity of illness as evidenced by a higher PRISM III and PELOD-2 scores at admission.

Organ dysfunction is common in scrub typhus. The mortality is proportionate to the severity and number of organ systems involved [8]. An elevated SOFA score has been independently associated with mortality in adult studies [8]. In our cohort, the median (IQR) PELOD2 scores at admission was 2 (2, 5) among survivors as compared to 7 (7, 9) in nonsurvivors.

Ferritin is being used as a marker to determine the body iron stores since 1970s [22]. Ever since, the focus on ferritin has diversified with its role in inflammation, infection, and malignancy. Surprisingly, our understanding on its mechanisms in different diseases is still enigmatic despite 5 decades of clinical research. Despite the mention in 2004 HLH criteria, elevation of ferritin greater than 500 μg/L could still be nonspecific in infections. In our study too, hyperferritinemia was seen in 72.6%, reiterating its function as an acute phase reactant to infection. More than half had mild elevation, but severe hyperferritinemia was seen only in 10% of children. Be it changing epidemiology or increased recognition, hyperferritinemia as a syndrome is increasingly diagnosed in critically ill children [23]. The pathogenesis of hyperferritinemia in sepsis/systemic inflammation continues to evolve in recent literature [24]. With growing evidence, ferritin is now believed to play a key role in normal host response to infection and immunomodulation rather than a nonspecific marker with sole function of sequestrating iron from the circulation [25, 26].

Illness-appropriate response in ferritin is believed to correlate with favorable outcome. Both high and very low responses were shown to be associated with increased mortality in patients with sepsis. Garcia et al. observed a mortality of 23% and 58% when ferritin values were < 200 μg/L and > 500 μg/L respectively. However, patients with ferritin between 200 and 500 had only 9% mortality [13]. Bennett et al. observed a stepwise increase in mortality risk in hospitalized children with ferritin ≥ 1000 μg/L and ≥ 3000 μg/L [27].

It has been highlighted that iron may not serve as an essential nutrient for intracellular obligate bacteria like Chlamydia and Rickettsial species. These organisms tend to grow in an iron-deficient media to avoid excessive free iron stress and also do not have functional homology sequences to siderophore synthesis enzymes in their genomes [28, 29]. Ellison et al. in their experimental study supported this hypothesis that there is minimal transcriptional response after iron depletion in rickettsial culture exposed to iron chelator 24 h after in vitro treatment [30]. Moreover, it is observed that *Orientia tsutsugamushi* infect human monocytic cells and increases expression of interferon type-1 genes resulting in a M1 type of monocyte-macrophage phenotype which sequesters iron within macrophages [31].

With this knowledge, it is likely that in individuals who have relatively normal iron stores, rickettsial infection would trigger the M1 macrophage phenotype response resulting in transient early hyperferritinemia due to macrophage sequestration of iron and increased transcription of ferritin gene as acute phase reactant. This response also leads to better interferon and pro-inflammatory cytokine secretion by macrophage resulting in better control of infection and a favorable outcome. However, those with low or absent stores can have a delayed elevation of ferritin. This when compounded by multiple other host factors such as secondary macrophage activation and organ dysfunction in late presenters could explain the high mortality in this subgroup. Also, it is logical to appreciate that the higher mortality is strongly linked to the delay in receiving specific antimicrobial therapy in the late presenters.

Four children (5.5%) were treated for secondary HLH, of whom 2 survived. Diagnosis of HLH is often challenging in scrub typhus as children would present with fever, cytopenia and organomegaly at the outset. Ferritin elevation > 500 μg/L can be very nonspecific as seen in our cohort. Secondary HLH may not require aggressive management when compared to primary HLH. Demirkol et al. showed 100% survival in children with hyperferritinemic sepsis MODS/MAS/HLH who were treated with methylprednisolone, IVIG, and plasma exchange, whereas those treated with the HLH-94 protocol using chemotherapy and dexamethasone had only 50% survival as all deaths occurred from overwhelming sepsis [15]. Our cohort received IvIg as first line; steroids were added in 2 children.

Our study has some important strengths. This is one of the largest prospective pediatric studies on scrub typhus evaluating the prevalence of hyperferritinemia. We included children with laboratory-confirmed scrub typhus and followed them up till 28 days with daily evaluation of organ dysfunction. However, a few limitations need mention. Our cohort had greater proportion of severe scrub typhus cases which could have led to increase in the prevalence of hyperferritinemia. The possibility of selection bias due to the stringent inclusion criteria that included features of organ dysfunction to increase the specificity of the diagnosis could not be negated. Children presented to emergency short stay area with less severe disease were not included that could potentially have overestimated the prevalence of hyperferritinemia. Majority of the patients were only IgM positive but PCR negative. The seasonal presentation, suggestive clinical syndrome along with IgM positivity, adds weight to the diagnosis of scrub typhus although it is not confirmatory. Serial ferritin measurements and its trend with respect to clinical course and treatment may give more answers regarding the pathogenesis.

## Conclusions

Hyperferritinemia is common in scrub typhus but it did not predict clinical outcomes. Organ dysfunction scores were found to be better predictors of mortality than ferritin.

## Data Availability

The datasets used and/or analyzed during the current study are available from the corresponding author on reasonable request.

## Abbreviations

AKI: Acute kidney injury
ARDS: Acute respiratory distress syndrome
DIC: Disseminated intravascular coagulation
ELISA: Enzyme-linked immunosorbent assay
IvIg: Intravenous immunoglobulin
KDIGO: Kidney Diseases Improving Global Outcomes
MODS: Multiorgan dysfunction syndrome
PCR: Polymerase chain reaction
PELOD: Pediatric Logistic Organ Dysfunction
PICU: Pediatric intensive care unit
PRISM: Pediatric risk of mortality
VIS: Vasoactive inotrope score
